# An easy and practical guide for imaging infection/inflammation by [^18^F]FDG PET/CT

**DOI:** 10.1007/s40336-021-00435-y

**Published:** 2021-06-01

**Authors:** Alberto Signore, Massimiliano Casali, Chiara Lauri

**Affiliations:** 1 Nuclear Medicine Unit, Department of Medical-Surgical Sciences and of Translational Medicine, “Sapienza” University of Rome, 00161 Rome, Italy; 2 Nuclear Medicine Unit, Oncology and High Technology Department, S. Maria Nuova Hospital, AUSL-IRCCS of Reggio Emilia, 42123 Reggio Emilia, Italy; 3 Nuclear Medicine Unit, Department of Medical-Surgical Sciences and of Translational Medicine, Ospedale S. Andrea, Via di Grottarossa 1035, 00189 Rome, Italy

**Keywords:** [^18^F]FDG PET/CT, Inflammation, Infection, Imaging, Interpretation criteria

## Abstract

**Aim:**

The aim of this mini-review was to summarize the role of positron emission tomography/computed tomography (PET/CT) with ^18^Fluorine-fluorodeoxyglucose ([^18^F]FDG) in inflammatory and infective processes, based on the published scientific evidence.

**Methods:**

We analysed clinical indications, patient preparation, image acquisition protocols, image interpretation, pitfalls and how to make the report of cardio-vascular diseases, musculoskeletal diseases and other inflammatory and infective systemic diseases.

Results of this analysis are shown in practical tables, easy to understand for daily routine consultation.

**Conclusions:**

Despite [^18^F]FDG is currently used in several inflammatory and infective diseases, standardized interpretation criteria are still needed in most cases. It is, therefore, foreseen the execution of multicentre clinical studies that, by adopting the same acquisition and interpretation criteria, may contribute to the standardization of this imaging modality.

## Introduction

The diagnosis of an infection by means of imaging modalities mainly relies on the possibility to exclude aseptic inflammation due to degenerative process, or autoimmune/allergic reactions or simply irritative causes. Several radiological and Nuclear Medicine procedures are, therefore, involved, in the search of which modality is more accurate in which clinical setting.

From the Nuclear Medicine point-of-view, this challenge to differentiate an infection from a sterile inflammation, has led to the production of hundreds of different radiopharmaceuticals that have open new ways to the possibility to specifically image the underlying process from a molecular point of view [1, 2].

Radiolabelled antibiotics [3, 4] or glucose derived sugars [5–8] have shown the potential to image bacteria, and, on the other hand, radiolabelled cytokines [9] or monoclonal antibodies [10] open the possibility to image different white blood cell subsets for histopathological characterization in vivo of the inflammatory/infective process.

Despite this enthusiastic output of new radiopharmaceuticals, the scintigraphy with radiolabelled white blood cells (WBCs), developed in early 1970 [11, 12], remains the Nuclear Medicine modality of choice for routine and accurate diagnosis of infection. Over the years, we learned that this technique strictly relies on the application of precise labelling modalities, image acquisition protocol and interpretation criteria that have been published as guidelines by the European Association of Nuclear Medicine (EANM) [13–18].

In the last two decades, given the increasing availability and application of positron emission tomography/computed tomography (PET/CT) with ^18^Fluorine-fluorodeoxyglucose ([^18^F]FDG) in several clinical contexts, infection and inflammation have also been extensively studied [19].

The great sensitivity of [^18^F]FDG, together with the high quality of images provided by new generation tomographs, suggest the use of this modality for both diagnostic and follow-up purposes [19].

Nevertheless, well-standardized interpretation criteria, as it has been done for radiolabelled WBC scintigraphy, still do not exist for many infective or inflammatory disorders, thus resulting in different approaches adopted by each centre, and, most important, in a wide variability of reported accuracies of this modality that do not allow to make a direct comparison of different studies.

The need of well standardized protocols for acquisition and interpretation of [^18^F]FDG PET/CT images in this field, has become essential amongst the Nuclear Medicine community, as demonstrated by the increasing number of consensus documents and proposed interpretation criteria that have been published, for example, for imaging of prosthetic joint infections [20–24], diabetic foot osteomyelitis [25–27], cardiovascular inflammations and infections [18, 28–34], spondylodiscitis [35], inflammatory bowel diseases [36] and, more recently, for imaging with [^18^F]FDG by PET/Magnetic Resonance Imaging (MRI) [37, 38].

Nonetheless, the proposed interpretation criteria for [^18^F]FDG PET/CT imaging in many clinical indications still need to be universally validated.

## Purpose

This mini-review aims at providing an overview on the state of art of [^18^F]FDG PET/CT imaging in musculoskeletal infections, cardiovascular infections and inflammations, and systemic inflammatory and infective diseases with particular emphasis on image acquisition protocols and interpretation criteria.

## Methods

In this mini-review, we summarize the available procedural recommendations for [^18^F]FDG PET/CT imaging in several infective and inflammatory conditions as derived from the literature of the past 20 years. An accurate and detailed analysis of the role of [^18^F]FDG PET/CT in each specific indication, resulting from an expert consensus, will be provided in the following article of this Special Issue of Clinical and Translational Imaging. In particular, an extensive literature research has been carried on the role of [^18^F]FDG PET/CT in osteomyelitis, prosthetic joint infections, spondylodiscitis, diabetic foot infections, infective endocarditis (both native an prosthetic valve endocarditis), cardiac implantable electronic devices infection, left ventricular assist device-associated infections, vascular graft infections, large vessel vasculitis, cardiac sarcoidosis, fever and inflammation of unknown origin, systemic sarcoidosis, inflammatory bowel disease, retroperitoneal fibrosis, fungal infections, tuberculosis and SARS CoV-2 infection.

In particular, each topic was summarized according to the following scheme:

- Clinical indications: gives an overview of the specific indications for the execution of [^18^F]FDG PET/CT in the diagnostic setting, for therapy evaluation or follow-up.
- Patient preparation: describes specific protocols, when required, that need to be adopted to increase the accuracy of this modality in detecting a specific disease.
- Imaging protocol: explains those specific acquisition protocols used for inflammatory and infective disease, when available.
- Interpretation criteria: provides a panoramic overview of recently published interpretation criteria of [^18^F]FDG PET/CT imaging in each specific disease.
- Possible pitfalls: this section summarizes the most frequently observed pitfalls and artefacts that need to be considered for a correct interpretation of the scan.
- Final report: describes how to report the exam (in addition to demographic data and technical information of the scan, type of tomograph, body weight and administered dose) with a focus on essential parts of the report. Time between injection and image acquisition should always be included in the report since it could be particularly useful for both long-term follow-up and therapy evaluation studies when SUV_max_ are compared.

## Results

Results are summarized in easy to read tables, aiming at proving a useful tool in daily practice (Tables 1, 2, 3, and 4).

**Table 1:** Summary table on [^18^F]FDG PET/CT imaging in cardiovascular infections/inflammations

Disease	Clinical indication	Patient preparation	Imaging protocol	Interpretation criteria	Pitfalls	Final report
Large vessel vasculitis	Diagnosis Therapy assessment	According to EANM/ SNMMI/PIG procedural recommendations	Whole body acquisitions (60’ after i.v. injection of 2–3 MBq/Kg of [^18^F]FDG) Late segmental acquisitions (90’-120’ p.i.) of suspected area	Qualitative analysis (1) Location Aorta and its major branches (2) Pattern Linear/segmental uptake large vessel vasculitis;focal uptake: atherosclerotic plaque (3) Intensity of uptake: Grade 0: no uptake; Grade I: less than liver; Grade II: similar to the liver; Grade III: higher than liver Grade ≥ II: large vessel vasculitis Semi-qualitative analysis Limited value for SUV_max_ or TVS	Steroid treatment could reduce accuracy FP results in in atherosclerosis	Presence/absence of vascular uptake Pattern of uptake Location and extent Intensity of uptake Comparison with previous [^18^F]FDG PET/CT if performed Time between injection and image acquisition (in order to better compare SUV_max_ of basal and FU studies)
Vascular graft infections	Identification of infection and evaluation of its extent Identification of septic embolism Therapy assessment	According to EANM/SNMMI procedural guidelines EANM/EACVI procedural recommendations on 4Is CV imaging	Whole body acquisitions (60’ after i.v. injection of 2.5–5.0 MBq/Kg of [^18^F]FDG) Steps: 1.5–3 min for bed position; Late segmental acquisitions (90’-120’ p.i.) of suspected area Administration of iodinated contrast may be useful to obtain a diagnostic CT scan	Qualitative analysis (1) Location Aorta and its major branches or peripheral grafts (2) Pattern Intense and focal and uptake, with dotted configuration: graft infection Mild and homogeneous uptake: non-infected graft (3) Intensity of uptake: Grade 0 (similar to the background): no infection; Grade I (similar to inactive muscles and fat): low [^18^F]FDG uptake; Grade II (≥ than inactive muscles and fat): moderate [^18^F]FDG uptake; Grade III (≤ than the physiologic urinary uptake by the bladder): strong [^18^F]FDG uptake; Grade IV (comparable with bladder uptake): very strong [^18^F]FDG uptake Focal uptake + Grade > II: vascular graft infections Semi-qualitative analysis Limited value for SUV_max_ or T/B ratios	Physiologic [^18^F]FDG uptake due to post-surgical inflammation; Venous thrombosis; Vasculitis; Retroperitoneal fibrosis; [^18^F]FDG-avid processes that are close to the graft	PET assessment Description of pattern and intensity; Location; Evaluation of extent of uptake; Description of eventual septic emboli CT assessment Description of graft’s border (regular vs irregular); Evaluation of other radiologic signs of infection (graft dislocation, presence of gas/fluid collections) Comparison with previous [^18^F]FDG PET/CT if performed; Time between injection and image acquisition (in order to better compare SUV_max_ of basal and FU studies)
Infective endocarditis	Suspected PVE; Identification of septic embolisms, mycotic aneurysms, spread of infection, POE in both PVE and NVE	According to EANM/EACVI procedural recommendations: High-fat–enriched diet lacking carbohydrates for 12–24 h prior to the scan; Fasting: 12–18 h; (optional) iv heparin of 50 IU/kg 15 min prior to [^18^F]FDG injection	Whole body acquisitions (60’-90’after i.v. injection of 2.5–5.0 MBq/Kg of [^18^F]FDG) Steps: 2 min for bed position; Optional: gated PET/CTA	Qualitative analysis (1) Location Intravalvular/valvular/ perivalvular (2) Pattern focal and non homogeneous: infection (3) Intensity of uptake high uptake: infection Semi-qualitative analysis Limited value for SUV_max_ or prosthesis/background ratios	Incomplete myocardial suppression of [^18^F]FDG; Lipomatous hypertrophy of the interatrial septum; [^18^F]FDG-avid processes close to the graft but not involving the device; Post-surgical sterile inflammation; Primary cardiac tumours or metastasis; Libman-Sacks endocarditis	Typical findings Presence of focal, heterogeneous, valvular/peri-valvular [^18^F]FDG uptake persisting on NAC images; High [^18^F]FDG signal in the absence of prior use of surgical adhesives; Presence of focal [^18^F]FDG uptake in organs with low background uptake: septic embolism, mycotic aneurysms or POE Atypical findings Diffuse, homogeneous, valvular [^18^F]FDG uptake that is absent on NAC images; Low [^18^F]FDG signal Comparison with previous [^18^F]FDG PET/CT if performed; Time between injection and image acquisition (in order to better compare SUV_max_ of basal and FU studies)
Cardiac implantable electronic device infection	Suspected cardiac implantable electronic device infection; Definition of the extent of infection; Positive blood culture in a patient with cardiac implantable electronic device	Same protocol described for infective endocarditis	Same protocol described for infective endocarditis; Late PET acquisitions might be useful in case of persistent high blood signal on PET images acquired at 1 h p.i	Qualitative analysis (1) Location Pocket/generator (superficial or deep) Leads (intravascular or intracardiac portion) (2) Pattern Focal or linear signal persisting on NAC images: infection (3) Intensity of uptake High uptake: infection Semi-qualitative analysis Limited value for SUV_max_ or T/B ratios	Same pitfalls described for infective endocarditis; Moderate uptake can be found up to 2 months after cardiac implantable electronic device implantation	Focal or linear uptake located on or alongside a lead and persisting on NAC images: infection; Multiple focal spots in the lungs: septic pulmonary emboli; Describe POE; Comparison with previous [^18^F]FDG PET/CT if performed; Time between injection and image acquisition (in order to better compare SUV_max_ of basal and FU studies)
Left ventricular assist device associated infections	Suspected left ventricular assist device associated infections; Evaluation of the extent; Positive blood culture in a patient with left ventricular assist device	Same protocol described for infective endocarditis	Same protocol described for infective endocarditis	Qualitative analysis (1) Location Driveline exit site/ driveline within the subcutaneous tissue/pump/inflow cannula/outflow cannula (2) Pattern Focal or linear signal persisting on NAC images: infection (3) Intensity of uptake High uptake: infection Semi-qualitative analysis Limited value for SUV_max_ or T/B ratios	The analysis of the FDG signal in the pump and cannula are more complex because of the artifacts caused by the device	Presence/absence of uptake Pattern description and location Extent Intensity of uptake The persistence of [^18^F]FDG uptake on NAC and its association with infiltration around the pump on the non-enhanced CT: infection; Comparison with previous [^18^F]FDG PET/CT if performed; Time between injection and image acquisition (in order to better compare SUV_max_ of basal and FU studies)
Cardiac sarcoidosis	Suspected cardiac sarcoidosis; Therapy assessment	Delaying steroid treatment initiation after the baseline scan is strongly recommended	Same protocol described for infective endocarditis	Qualitative analysis (1) Location Left or right cameras (2) Pattern No [^18^F]FDG uptake/diffuse (homogeneus) [^18^F]FDG uptake/ isolated [^18^F]FDG uptake on lateral wall uptake + normal perfusion + no LGE at CMR: No cardiac sarcoidosis; No [^18^F]FDG uptake + small perfusion defect + one focal area of LGE or Focal area of [^18^F]FDG uptake + normal perfusion + one focal area of LGE: possible cardiac sarcoidosis (50–90%); No [^18^F]FDG uptake + multiple non-contiguous areas of perfusion defect / + typical LGE or Focal [^18^F]FDG uptake/focal on diffuse [^18^F]FDG uptake + resting perfusion defect + typical LGE: probable cardiac sarcoidosis (50–90%) Focal area + extracardiac findings + normal perfusion + typical LGE: active cardiac sarcoidosis (> 90%); Focal on diffuse [^18^F]FDG uptake + perfusion defect + typical LGE: active inflammation with scar; Focal area of [^18^F]FDG uptake in a normally perfused area + perfusion defect in another area + typical LGE: inactive scar + inflammation (or FP [^18^F]FDG uptake) in different segments (3) Intensity of uptake High uptake: higher probability of cardiac sarcoidosis Semi-qualitative analysis SUV_max_ is reliable for both diagnosis and therapy efficacy assessment	Same pitfalls previously described	Description of the findings for both qualitative and semi-quantitative point of view; Possible differential diagnosis; Comparison to previous ^18^F-FDG PET/CT, if performed; Time between injection and image acquisition (in order to better compare SUV_max_ of basal and FU studies)

*EANM* European Association of Nuclear Medicine, *SNMMI* Society of Nuclear Medicine and Molecular Imaging, *PIG* PET Interest Group, *i.v.* intra-venous, *MBq* Mega Bequerel, *Kg* Kilograms, *[*^*18*^*F]FDG* 18Fluorine fluorodeoxyglucose, *p.i.* post-injection, *SUV*_*max*_ standardized uptake value, *T/B* target/background, *FU* follow-up, *PVE* prosthetic valve endocarditis, *NVE* native valve endocarditis, *POE* portal of entry, *NAC* non-attenuated CT, *TVS* total vascular score, *FP* false positive, *CT* computed tomography, *PET/CT* positron emission tomography/computed tomography, *VGI* vascular graft infections, *CTA* computed tomography angiography, *LGE* late Gadolinium enhancement, *CMR* cardiac magnetic resonance

**Table 2:** Summary table on [^18^F]FDG PET/CT imaging in musculoskeletal infections/inflammations

Disease	Clinical indication	Patient preparation	Imaging protocol	Interpretation criteria	Pitfalls	Final report
Spinal Infections	Diagnosis of suspected primary or secondary spinal infections; Suspected recurrence; Evaluation of extent and complications; Evaluation of antibiotic efficacy	According to Joint EANM/ ESNR and ESCMID-endorsed consensus document	Whole body acquisitions (50–60’ after i.v. injection of 2.5–3 MBq/Kg of [^18^F]FDG)	Qualitative analysis (1) Location Vertebral body (2) Pattern Smooth and homogeneous uptake: no infection (3) Intensity of uptake Score 0 (no uptake): no infection; Score I (slightly increased uptake in the inter- or paravertebral region): no infection; Score II (clearly increased uptake with a linear or disciform pattern in the intervertebral space): discitis; Score III (Score II + involvement of ground or cover plate or both plates of the adjacent vertebrae): spondylodiscitis; Score IV (Score III + surrounding STs abscess): spondylodiscitis Semi-qualitative analysis ΔSUV_max_ between 25 and 43% could be useful for the assessment of therapy response	FP findings in Inflammatory or degenerative disc diseases; Bone tumours or metastases; Recent vertebral fractures; Post-surgical inflammation; FN findings in Low-virulence bacterial infections; Previous antimicrobial treatment; Epidural abscesses; Extensive arthrodesis	Presence/absence of lesions; Pattern of uptake; Location; Extent; Intensity of uptake; Comparison with previous [^18^F]FDG PET/CT if performed; Time between injection and image acquisition (in order to better compare SUV_max_ of basal and FU studies)
Diabetic foot infections	Detection of infection (mainly in forefoot) and evaluation of its extent; DD between osteomyelitis, soft tissue infections and Charcot; Therapy monitoring and follow-up	According to EANM/SNMMI procedural guidelines	Whole body or, preferably, segmental acquisitions (60’ after i.v. injection of 2.5–5.0 MBq/Kg of [^18^F]FDG)	Qualitative analysis (1) Location in forefoot osteomyelitis, mandatory correlation of FDG uptake with CT abnormalities in bone in mid-hindfoot osteomyelitis, necessary correlation with WBC scan and colloid scan (2) Pattern: focal/diffuse uptake higher than contralateral clearly involving the bone: osteomyelitis; focal/diffuse uptake detectable only on STs: soft tissue infections; diffuse uptake involving mid-hindfoot and associated to disruption of bony architecture on CT: suggestive of Charcot Semi-qualitative analysis Limited value for SUV_max_ or T/B ratios	Pre-existing orthopaedic comorbidities (fractures/ arthrosis/arthritis…); Difficult to achieve and accurate DD between non infected Charcot and Charcot with super-imposed infection	Presence/absence of lesions; Pattern of uptake; Location; Extent; Intensity of uptake; Evaluation of CT component; Comparison to previous [^18^F]FDG PET/CT, if performed; Time between injection and image acquisition (in order to better compare SUV_max_ of basal and FU studies)
Osteomyelitis and prosthetic joint infections	Diagnosis of chronic osteomyelitis, destructive septic arthritis, prosthetic joint infections, infected fractures; Therapy monitoring	According to EANM/SNMMI procedural guidelines	Whole body or segmental acquisitions (60’ after i.v. injection of 2.5–5.0 MBq/Kg of [^18^F]FDG)	Qualitative analysis For prosthetic joint infections, the most important criterion seems to be the location of the uptake rather than the pattern or SUV_max_ Several interpretation criteria have been proposed but none has been universally accepted Peripheral bone osteomyelitis (1) Location Increased uptake higher than Contralateral clearly involving the bone: osteomyelitis (2) Pattern: Focal/linear/diffuse uptake: focal uptake clearly involving a bone segment: osteomyelitis; Semi-qualitative analysis Limited value for SUV_max_ or T/B ratios	Difficult to achieve and accurate DD between aseptic prosthetic loosening, infection, inflammation, degenerative changes and malignancy; Recent fractures and presence of metallic hardware may decrease the accuracy of [^18^F]FDG PET/CT	Presence/absence of uptake; Pattern of uptake; Location; Extent; Intensity of uptake; Evaluation of CT component; Comparison to previous [^18^F]FDG PET/CT, if performed; Time between injection and image acquisition (in order to better compare SUV_max_ of basal and FU studies)

*EANM* European Association of Nuclear Medicine, *ESNR* European Society of Neuroradiology, *ESCMID* European Society of Clinical Microbiology and Infectious Disease, *i.v.* intra-venous, *MBq* Mega Bequerel, *Kg* Kilograms, *[*^*18*^*F]FDG* 18Fluorine fluorodeoxyglucose, *p.i.* post-injection, *SUV*_*max*_ standardized uptake value, *T/B* target/background, *ΔSUV*_*max*_ SUV_max_ before treatment- SUV_max_ after treatment, *FP*: false positive, *FN* false negative, *FU* follow-up, *DD* differential diagnosis, *STs* soft tissues, *SNMMI* Society of Nuclear Medicine and Molecular Imaging, *CT* computed tomography, *PET/CT* positron emission tomography/computed tomography, *BPI* bone/prosthesis interface

**Table 3:** Summary table on [^18^F]FDG PET/CT imaging in systemic inflammations

Disease	Clinical indication	Patient preparation	Imaging protocol	Interpretation criteria	Pitfalls	Final report
Retroperitoneal Fibrosis	Diagnosis; Evaluation of disease during/after treatment in patients with normal inflammatory markers and stable residual mass; Evaluation of correct time to proceed to ureteral stent removal; Discrimination between active and residual fibrotic tissue	According to EANM/SNMMI procedural guidelines	Whole body acquisitions (60’ after i.v. injection of 2.5–3 MBq/Kg of [^18^F]FDG)	Qualitative analysis (1) Location Anatomical description of pathologic tissue and its relationships with vascular and ureteral structures (2) Pattern diffuse, segmental, focal (3) Intensity of uptake Score 0: no uptake Score I: uptake < liver; Score II: uptake similar to liver; Score III: uptake > liver Semi-quantitative analysis Limited value for SUV_max_ or T/B ratios	FP findings in Beam-hardening artifact; Diffuse aortic calcifications FN findings under steroid or immunosuppressive therapy	Presence/absence of uptake; Pattern of uptake; Location; Extent; Intensity of uptake; Possible DD; Comparison with previous [^18^F]FDG PET/CT if performed; Time between injection and image acquisition (in order to better compare SUV_max_ of basal and FU studies)
Fever of Unknown Origin / Inflammation of Unknown Origin	Evaluation of unknown inflammatory, infective or neoplastic sites; Guide biopsy; Evaluation of therapy efficacy	According to EANM/SNMMI procedural guidelines	Whole body acquisitions (60’ after i.v. injection of 2.5–3 MBq/Kg of [^18^F]FDG)	Qualitative analysis Based on the identification of all sites of pathological tracer uptake	[^18^F]FDG is not able to discriminate between infection and inflammation; FN findings in patient under antibiotic treatment or steroid/immunosuppressive therapy FP findings in neoplastic tissues	Presence/absence of uptake; Pattern of uptake; Location; Extent; Intensity of uptake; Possible DD; Comparison with previous [^18^F]FDG PET/CT if performed; Time between injection and image acquisition (in order to better compare SUV_max_ of basal and FU studies)
Inflammatory Bowel Diseases	Diagnosis in patients with suspected inflammatory bowel diseases in equivocal cases Intestinal and extra-intestinal disease assessment; Evaluation of complications; Early evaluation of therapy efficacy Follow-up and monitoring disease evolution	According to EANM/SNMMI procedural guidelines	Whole body acquisitions 60’ after i.v. injection of 2.5–3 MBq/Kg of [^18^F]FDG)	Qualitative analysis (1) Location Crohn’s Disease: any segment of GI tract; Ulcerative Colitis: mainly involves rectum with a possible extent to proximal parts (2) Pattern diffuse, segmental, focal (3) Intensity of uptake: Diffuse and mild glucose uptake in bowel: negative for inflammatory bowel diseases; Segmental and significant increased uptake in the intestinal tract: positive for inflammatory bowel diseases; Semi-quantitative analysis Bowel SUV_max_ > than liver is suggestive for inflammatory bowel diseases However, no defined SUV_max_ cut-off has been identified	FP findings in: Diabetic patients assuming hypoglycemic oral therapy; Diverticulitis; Infectious colitis; Malignancies FN findings in: Disease with a low grade activity; Recent administration of high dose of corticosteroid	Presence of increased glucose uptake in bowel segments and/or in extra-intestinal sites, Pattern of uptake Extent Intensity of uptake; Possible DD; Comparison with previous [^18^F]FDG PET/CT if performed; Time between injection and image acquisition (in order to better compare SUV_max_ of basal and FU studies)
Systemic sarcoidosis and tubercolosis	Evaluation of disease activity and extent; DD between reversible granuloma from irreversible fibrosis; Diagnosis of occult disease; Evaluation of treatment response; Guide biopsy	According to EANM/SNMMI procedural guidelines	Whole body acquisitions (from vertex to distal extremities of the lower limbs, 60’ after i.v. injection of 2.5–3 MBq/Kg of [^18^F]FDG)	Qualitative analysis Description of lymph nodes (lambda sign), pulmonary, pleural, lacrimal and a salivary glands, brain, musculoskeletal and brain involvement; For assessing myocardial involvement, see Table 1 Semi-quantitative analysis Limited value for SUV_max_ or T/B ratios	[^18^F]FDG is not able to achieve an accurate DD between infections, inflammation and Malignancies (lymphomas)	Description of any site of increased glucose uptake, Pattern of uptake distribution Intensity of uptake; Possible DD; Comparison with previous [^18^F]FDG PET/CT if performed; Time between injection and image acquisition (in order to better compare SUV_max_ of basal and FU studies)

*EANM* European Association of Nuclear Medicine, *i.v.* intra-venous, *MBq* Mega Bequerel, *Kg* Kilograms, *[*^*18*^*F]FDG* 18Fluorine fluorodeoxyglucose, *p.i.* post-injection, *SUV*_*max*_ standardized uptake value, *T/B* target/background, DD differential diagnosis, *SS* systemic sarcoidosis, *SNMMI* Society of Nuclear Medicine and Molecular Imaging, *CT* computed tomography, *PET/CT* positron emission tomography/computed tomography, *FP* false positive, *FN* false negative, *FU* follow-up, *GI* gastro-intestinal

**Table 4:** Summary table on [^18^F]FDG PET/CT imaging in fungal and viral infections

Disease	Clinical indication	Patient preparation	Imaging protocol	Interpretation criteria	Pitfalls	Final report
Invasive Fungal Infections	To identify clinically occult and disseminated invasive fungal infections in immune-compromised and HIV-positive patients when CT is non-contributory; To monitor treatment response; To diagnose HIV-related opportunistic infections, associated neoplasms, and Castleman’s disease; To monitor response to HAART in HIV-positive patients	To avoid the use of non-steroidal anti-inflammatory drugs, glucocorticoids or immunosuppressive agents; According to EANM/SNMMI procedural guidelines	Whole body acquisitions (50–60’ after i.v. injection of 4–5 MBq/Kg of [^18^F]FDG); Additional acquisition of lower limbs (1–3 min/bed) could be helpful in selected patients	Qualitative analysis (1) Pattern: Focal uptake: strongly suggestive for invasive fungal infections; Diffuse uptake in subcutaneous fat: could be related to HIV-associated lipodystrophy syndrome (2) Intensity of uptake: Splenic uptake > hepatic uptake: earlier stages of HIV with a lymphomatous involvement of the spleen; Hypermetabolism of basal ganglia and globally reduced cortical uptake: HIV patients with subclinical neurologic dysfunction; Increased uptake in bone marrow, spleen and lymph nodes: immune reconstitution inflammatory syndrome Semi-qualitative analysis Limited role for SUV_max_	FP findings in Neoplasms; Other infections; Benign hypermetabolic lymph nodes in HIV patients could mimic lymphoma FN findings in: Small lesion size; Low metabolic rate; Ongoing steroid treatment	Presence/absence of lesions; Pattern of uptake; Location; Extent; Intensity of uptake; Possible DD; Comparison with previous [^18^F]FDG PET/CT if performed; Time between injection and image acquisition (in order to better compare SUV_max_ of basal and FU studies)
SARS-CoV2	Detection of lung inflammatory status and evaluation of its extent; Monitoring inflammation, its progression and treatment outcomes	According to EANM/SNMMI procedural guidelines	Whole body acquisitions (60’ after i.v. injection of 2.5–5.0 MBq/Kg of [^18^F]FDG)	Qualitative analysis (1) Location Involved lung (right and/or left), lobes and segments, mediastinal lymph nodes (2) Pattern Usually diffuse uptake on ground-glass/consolidative area detected by CT Semi-qualitative analysis Limited value for SUV_max_	Drug-induced interstitial pneumonia; Pneumonia of other etiology	Presence/absence of uptake; Pattern of uptake; Location; Extent; Intensity of uptake; Evaluation of CT component; Possible DD; Comparison with previous [^18^F]FDG PET/CT if performed; Comparison to previous ^18^F-FDG PET/CT, if performed; Time between injection and image acquisition (in order to better compare SUV_max_ of basal and FU studies)

*HIV* human immunodeficiency virus, *HAART* highly active anti-retroviral therapy, *EANM* European Association of Nuclear Medicine, *SNMMI* Society of Nuclear Medicine and Molecular Imaging, *i.v.* intra-venous, *MBq* Mega Bequerel, Kg Kilograms, *[*^*18*^*F]FDG* 18Fluorine fluorodeoxyglucose, p.i. post-injection, SUV_max_ standardized uptake value, *FP* false positive, *FN* false negative, *FU* follow-up, *DD* differential diagnosis, S*ARS-CoV2* severe acute respiratory syndrome coronavirus 2, *CT* computed tomography, *PET/CT* positron emission tomography/computed tomography

It emerges that standardized protocols for patient preparation, image acquisition and interpretation criteria exist only for very limited clinical indications in the field of infection and inflammation and, in particular, for infective endocarditis, cardiac implantable devices infections, left ventricular assist device-associated infections, cardiac sarcoidosis, large vessel vasculitis and spondylodiscitis. For all other clinical indications, the recommendations for patient preparation and the acquisition protocols, commonly adopted for oncologic studies, are currently applied. As far as image interpretation is concerned, several criteria have been proposed for vascular graft infections, osteomyelitis, diabetic foot infections, prosthetic joint infections, and systemic infections/inflammations, but they still need to be validated in larger multicentre studies being the reported diagnostic accuracy of single centre studies, extremely variable and generally lower than the diagnostic accuracy of WBC scintigraphy [20, 27].

## Conclusions

In summary, this article and the following, published in this journal, provide a useful tool for identifying several patterns of [^18^F]FDG uptake able to discriminate between an infection and a sterile inflammation aiming at increasing the specificity and the accuracy of this radiopharmaceutical. This may have a great clinical impact on the management of each specific disease, may help to smooth the wide heterogeneity that is still evident in literature and will lay the basis for future comparative studies.

The definition of disease-specific acquisition protocols is warranted to increase the specificity and accuracy of this imaging modality. Moreover, it is mandatory, that the definition of precise and standardized interpretation criteria for [^18^F]FDG PET/CT imaging in different infective or inflammatory disorders need to be adopted and shared by several institutions and validated in large, possibly multicentre, studies.

## Teaching points

- [^18^F]FDG has been proposed for the study of several inflammatory and infective diseases.
- Standardized acquisition and interpretation protocols exist for infective endocarditis and cardiac implantable electronic devices infections, cardiac sarcoidosis, large vessel vasculitis, as well as spondylodiscitis.
- Multicentre studies are needed to standardize the use of [^18^F]FDG in other inflammatory/infective diseases.
- Tables presented in this article can be used as a base for future studies.
